# Genomic Landscape of Young-Onset Bladder Cancer and Its Prognostic Implications on Adult Bladder Cancer

**DOI:** 10.3390/cancers12020307

**Published:** 2020-01-28

**Authors:** Sun-Wha Im, Chang Ohk Sung, Kun Suk Kim, Nam Hoon Cho, Young Min Kim, Ghee Young Kwon, Kyung Chul Moon, Song-Yi Choi, Jae Sung Lim, Yeong Jin Choi, Soo Jin Jung, So Dug Lim, Sung Hyun Paick, Ok-Jun Lee, Ho Won Kang, Seo Hee Rha, Hee Sang Hwang, Ja-Min Park, Sun Young Yoon, Jeesoo Chae, Jaeyong Choi, Jong-Il Kim, Yong Mee Cho

**Affiliations:** 1 Genomic Medicine Institute, Medical Research Center, Seoul National University, Seoul 03080, Korea; first@snu.ac.kr; 2 Department of Pathology, Asan Medical Center, University of Ulsan College of Medicine, Seoul 05505, Korea; co.sung@amc.seoul.kr (C.O.S.); eldersage@empas.com (H.S.H.); 3 Department of Urology, Asan Medical Center, University of Ulsan College of Medicine, Seoul 05505, Korea; kskim2@amc.seoul.kr; 4 Department of Pathology, Yonsei Medical University College of Medicine, Seoul 03722, Korea; cho1988@yuhs.ac; 5 Department of Pathology, Ulsan University Hospital, University of Ulsan College of Medicine, Ulsan 44033, Korea; ymkpath@uuh.ulsan.kr; 6 Department of Pathology, Samsung Medical Center, Sungkyunkwan University School of Medicine, Seoul 06351, Korea; geeo@skku.edu; 7 Department of Pathology, Seoul National University College of Medicine, Seoul 03080, Korea; blue7270@snu.ac.kr; 8 Department of Pathology, Chungnam National University College of Medicine, Daejeon 35015, Korea; sychoi@cnu.ac.kr; 9 Department of Urology, Chungnam National University College of Medicine, Daejeon 35015, Korea; uro17@cnu.ac.kr; 10 Department of Hospital Pathology, The Catholic University of Korea, Seoul 06591, Korea; mdyjchoi@catholic.ac.kr; 11 Department of Pathology, Inje University Busan Paik Hospital, Busan, Seoul 47392, Korea; soojinmd@hanmail.net; 12 Department of Pathology, School of Medicine, Konkuk University, Seoul 05029, Korea; 13 Department of Urology, School of Medicine, Konkuk University, Seoul 05029, Korea; 20030010@kuh.ac.kr; 14 Department of Pathology, Chungbuk National University Hospital, Chungbuk National University College of Medicine, Cheongju 28644, Korea; ok5218@hanmail.net; 15 Department of Urology, Chungbuk National University Hospital, Chungbuk National University College of Medicine, Cheongju 28644, Korea; howon98@naver.com; 16 Department of Pathology, College of Medicine, Dong-A University, Busan 49201, Korea; shrha@dau.ac.kr; 17 Asan Institute of Life Science, Asan Medical Center, Seoul 05505, Korea; parkja09@naver.com (J.-M.P.); mysunyoung14@naver.com (S.Y.Y.); 18 Department of Biomedical Science, Seoul National University Graduate School, Seoul 03080, Korea; moverm0210@gmail.com (J.C.); mesnger12@gmail.com (J.C.); 19 Cancer Research Institute, Seoul National University, Seoul 03080, Korea

**Keywords:** FGFR3, HRAS, next generation sequencing, prognosis, young-onset bladder cancer

## Abstract

Due to the rare occurrence of young-onset bladder cancer (YBC), its genomic characteristics remain largely unknown. Twenty-nine biopsy-proven YBC cases were collected using a nation-wide search for bladder cancer diagnosed at 20 years or younger. Whole exome sequencing and RNA sequencing were carried out in 21 and 11 cases, respectively, and compared with those of adult bladder cancer (ABC) cases obtained from public databases. Almost all YBCs were low grade, non-invasive papillary tumors. YBC had a low mutation burden and less complex copy number alterations. All cases harbored putative driver mutations. Mutations were most commonly found in *HRAS* (10 cases), with a preference for exon 5. *FGFR3* gene fusions were noted with various partner genes (7 cases). The alterations on *HRAS* and *FGFR3* occurred in a mutually exclusive manner. Others included *KRAS* mutations (2 cases), chromosomes 4p and 10q arm-level deletions (1 case), and *ERCC2* mutation (1 case). There were no point mutations in *TP53* and *FGFR3*. The gene expression profiles of YBC were similar to those of the ABC group with good prognosis. None of the YBCs and ABCs with YBC-like mutations showed progression to muscle-invasive tumors. Our results suggest that bladder cancer with YBC-like mutations represents an indolent bladder tumor, regardless of age.

## 1. Introduction

Bladder cancer is the fourth most common cancer and the seventh leading cause of cancer-related death in men in developed countries. Bladder cancer usually affects elderly men, with a median age of 65 to 70 years at initial diagnosis [1,2,3]. Approximately 70 to 80% of patients present non-muscle-invasive bladder cancer (NMIBC), including non-invasive bladder cancer (NIBC) and superficially invasive urothelial tumors with no involvement of the muscularis propria. NMIBC is not life-threatening, but it has a frequent recurrence rate of 50 to 70%. NMIBC progresses to muscularis propria-invasive bladder cancer (MIBC) in 15 to 25% of cases, which has a high risk of distant metastases and death [1]; patients with NMIBC are subject to lifetime follow-ups with urine cytology and cystoscopy, which imposes significant suffering and medical costs [4]. 

Unlike the high occurrence of bladder cancer in adult populations, the occurrence of bladder cancer in patients with 20 years or younger is extremely rare, corresponding to 0.1%–0.4% of urothelial tumors [5]. The clinical understanding of young-onset bladder cancer (YBC) is limited, and the management strategy is primarily based on that of adult bladder cancer (ABC).

MIBC in adults is characterized by frequent somatic mutations, and *TP53* is the most commonly mutated gene [6]. In NMIBC, *KDM6A* and *FGFR3* are commonly mutated [7]. In addition, many research groups, including the University of North Carolina, MD Anderson, The Cancer Genome Atlas (TCGA), and Lund University have proposed molecular classifications of adult MIBC based on the global gene expression of as few as two to as many as 12 tiers [6,8,9,10,11,12]. A European multicenter prospective study (UROMOL) classified adult NMIBC into three molecular subclasses and suggested 117 gene classifiers [7]. In the UROMOL study, classes 1 and 3 were characterized as Ta pathway with good prognosis, and class 2 was characterized as carcinoma in situ (CIS) pathway with poor prognosis. Sjödahl et al. classified 308 bladder tumors including 213 NMIBC into five molecular subtypes with different survival rates [13]. Patschan et al. also classified 167 NMIBC (T1) into three molecular subtypes, based on immunohistochemical characteristics, and compared the progression risk among them [14]. A recent metacohort study used transcriptomic data to classify 2411 MIBC and NMIBC into six subtypes, which demonstrated different overall survival and molecular features [15].

Previous studies reported that genetic alterations frequently observed in ABCs were extremely rare in YBCs [16], which may be because only small numbers of YBC cases have been analyzed by a limited number of candidate gene analyses. Nevertheless, previous studies reported that *HRAS* mutation rates were relatively high, but *FGFR3* and *TP53* mutations were rare in this age group [17,18,19,20]. Here, we analyzed YBC molecular alterations in detail using whole exome sequencing (WES) and RNA sequencing, and we compared our results to the ABC results.

## 2. Results

### 2.1. Summary of Genomic and Clinicopathological Features of YBC

**Figure 1:**
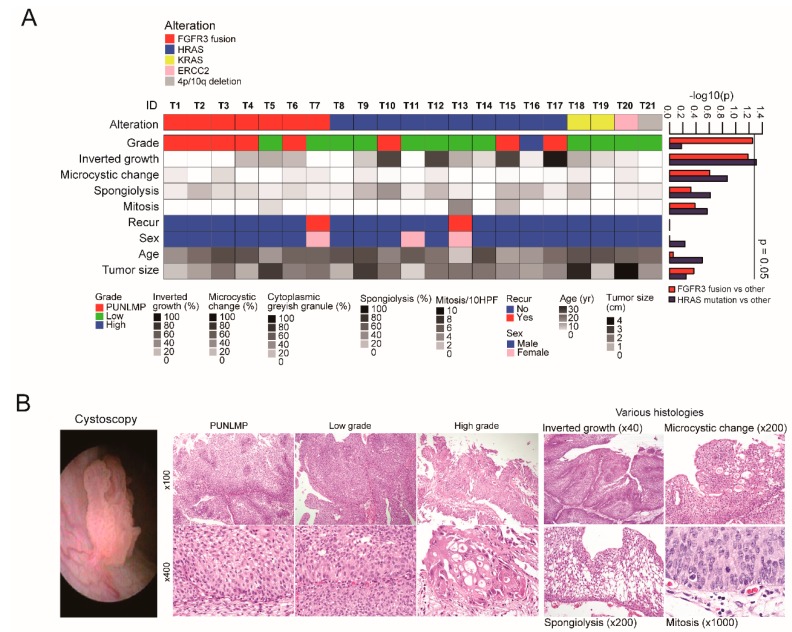
Characteristics of young-onset bladder cancer. (**A**) Summary of genetic alterations and correlations to clinicopathological features. (**B**) Representative images of different tumor grades (papillary urothelial neoplasm of low malignant potential (PUNLMP), low grade, and high grade) and histologic features of young-onset bladder cancer.

The clinical characteristics and putative driver genetic alterations of the YBC cases are presented in Figure 1 and Table S1. YBC was more common in males (M:F = 2.6:1), with a median age of 17 years (range: 5–20 years). In 28 cases that had available cystoscopic findings, tumors were all single masses with a median size of 1.9 cm (range: 0.5–5 cm). The tumors were papillary urothelial neoplasm of low malignant potential (PUNLMP, 11 cases) or low grade papillary urothelial carcinoma (17 cases). Only one case was high grade. Histologic features like inverted growth pattern, spongiolysis, microcysts, and mitosis were noted. All cases were non-invasive and characterized as early stage urothelial tumors. Except for four cases with a history of either neuroblastoma, epilepsy, Costello syndrome, or polycystic ovary syndrome with major depressive disorder, none had a remarkable past medical history. Neither smoking history nor family bladder cancer history was found in any case. The YBC cases were all alive during a median follow-up period of 62 months (range: 2–153 months). Three cases developed tumor recurrence with similar pathologic features. 

In the 21 cases with WES, the median number and frequency of nonsynonymous somatic mutations per sample were 15 and 0.3/Mb, respectively. Putative driver genetic alterations were found in all cases: *FGFR3* gene fusions in seven cases, *HRAS* mutations in 10 cases, *KRAS* mutations in two cases, arm-level deletion of chromosomes 4p and 10q in one case, and an *ERCC2* mutation in one case (Figure 1A). Tumors with an *FGFR3* fusion tended to be of PUNLMP in tumor grade (*p* = 0.056): five out of the seven cases (71.4%) with *FGFR3* fusion were of PUNLMP. The *HRAS* mutation was associated with inverted tumor growth (*p* = 0.0489, Figure 1B). 

### 2.2. FGFR3 Fusions with Various Partner Genes in YBC

**Figure 2:**
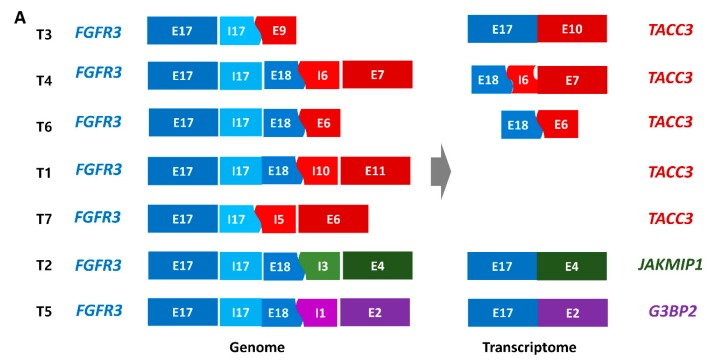

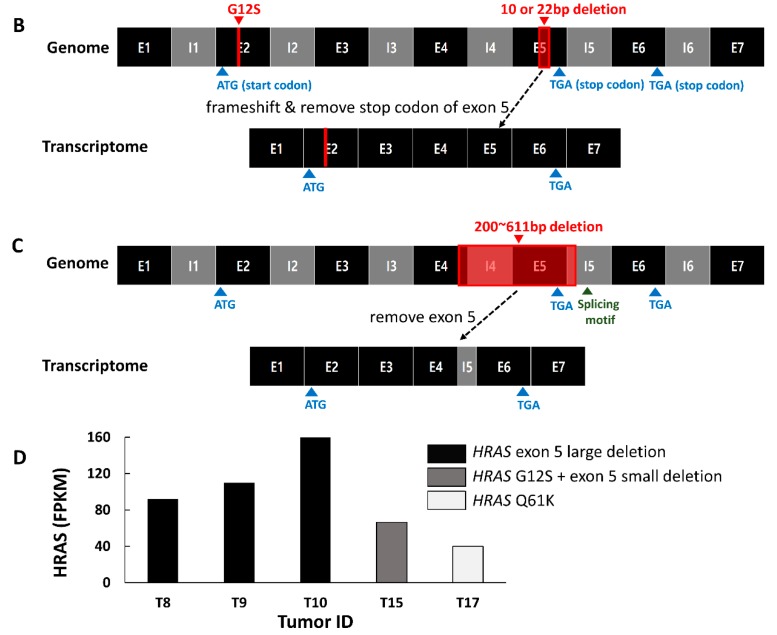
Genetic alterations of *FGFR3* and *HRAS* and their impact on gene expression. (**A**) Schematic description of genomic and transcriptomic fusion constructs of *FGFR3*. Small (**B**) or large (**C**) genomic deletion of exon 5 generated *HRAS* transcripts including exon 6. (**A–C**) E and I refer to exon and intron, respectively. (**D**) Gene expression of *HRAS* in 5 cases with *HRAS* mutations in young-onset bladder cancer.

Seven cases harbored chromosomal translocations that showed *FGFR3* gene fusion with three different partner genes: *TACC3* (n = 5)*, JAKMIP1* (n = 1), and *G3BP2* (n = 1) (Figure 2A). *FGFR3* commonly ended at its last intron (I17) or exon (E18) (ENST00000340107) and continued to various sites of the partner genes from that point. The translocations were confirmed using RNA sequencing (n = 5), Sanger sequencing (n = 6, Figure S1), or both (n = 5), except for one case whose additional sample was unavailable. When the fusion breakpoint was in the middle of the exon, it transcribed to RNA accordingly (T4, T6) or after skipping the exon (T2, T3, T5). In the T4 case, a part of a *TACC3* intron (I6) was included in the transcript and continued to the next exon in-frame. We found active expression of genes involved in fusion, and expression levels remarkably differed before and after the breakpoints, especially in the 3′ partner genes (*TACC3*, *JAKMIP1*, and *G3BP2*, Figure S2). 

### 2.3. Mutation Characteristics of YBC

Among the 10 cases with the *HRAS* (ENST00000493230) mutation, four cases had both nonsynonymous base substitutions (G12S) at exon 2 and a small deletion (10 or 22 bp) at exon 5 (Figure S3A and Figure 2B). One of the four cases was a patient with Costello syndrome with a germline G12S mutation, and the small deletion of exon 5 was a somatic event. The small deletions shifted the rest of the exon 5 reading frame and removed the stop codon of exon 5 to create a transcript leading to exon 6. Another four cases showed large (200 to 611 bp) deletions encompassing the terminal part of exon 4, intron 4, exon 5, and the beginning of intron 5 (Figure S3B and Figure 2C). The breakpoints of intron 5 were located near the 5′ splicing motif. The truncated exon 4 led to intron 5, met the splicing motif, broke at that point, and led to exon 6. The remaining two cases had a mutation in either G13R or Q61K. 

RNA sequencing showed that the *HRAS* expression level was the highest in the group with a large deletion, followed by the group with concurrent G12S and exon 5 small deletion, and the group with G13 or Q61 mutations only (Figure 2D), showing differential expressional regulation according to the *HRAS* mutation pattern.

Other mutations included a *KRAS* gene mutation that caused G12D and G12C in each case. One case harbored an *ERCC2* mutation causing M677I, which was located near its conserved helicase motif. 

### 2.4. Somatic Copy Number Alterations in YBC

A somatic copy number alteration (CNA) was identified in only three cases: one had a whole chromosome tetraploid and concurrent *FGFR3-TACC3* fusion (Figure S4A), and another had a 9p21 deletion and concurrent *KRAS* G12C mutation (Figure S4B). The 9p21 deletion is one of the most frequently reported somatic CNAs in bladder cancer, and it covers the *CDKN2A* gene. The remaining case had arm-level deletions of chromosomes 4p and 10q without any other driver mutations (Figure S4C). 

### 2.5. Gene Expression Signature Indicating Good Prognosis of YBC

The molecular classification of bladder cancer was attempted to complement the histopathological diagnosis and predict the prognosis. The transcriptomic characteristics of YBC were compared to those of the UROMOL study, which is the largest recent NMIBC transcriptomic analysis.

**Figure 3:**
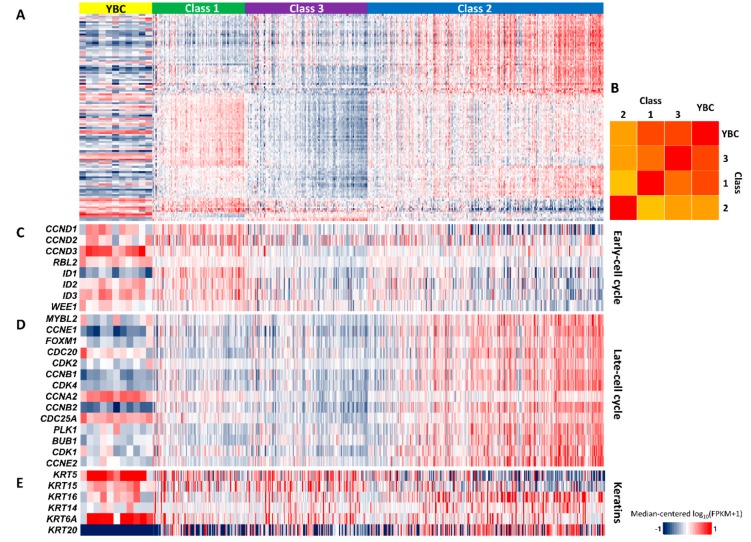
Transcriptomic characteristics of young-onset bladder cancer (YBC) compared to adult non-muscle-invasive bladder cancer (NMIBC) (UROMOL). Heatmap of 117 classifiers (**A**), early-cell cycle (**C**), late-cell cycle (**D**), and keratin (**E**) genes are presented in each panel. (**B**) Molecular distances between YBC and the three classes of UROMOL data based on 117 classifier genes are presented as Pearson correlation coefficients.

First, we determined that there was no noticeable batch effect in the principal component analysis, despite using different sequencing methods (Figure S5 and Figure 3A). When the median values of each gene in the group were taken as the representative value, the YBC samples showed features of classes 3 and 1 (Pearson similarity 0.34 and 0.32, respectively) with negative correlation to class 2 (Pearson similarity −0.49, Figure 3B). The YBC samples showed increased expression of early-cell cycle genes and decreased expression of late-cell cycle genes, corresponding to class 1 in the UROMOL study and considered indicators of good prognosis (Figure 3C,D) [7]. High *KRT20* and low *KRT5* expression, which represent class 2 in the UROMOL study and are regarded as a marker of poor prognosis, were inversely observed in YBC (Figure 3E) [21]. These molecular characteristics are consistent with good YBC prognosis. 

### 2.6. Differences in Genetic Alteration Pattern Between YBC and ABC

**Figure 4:**
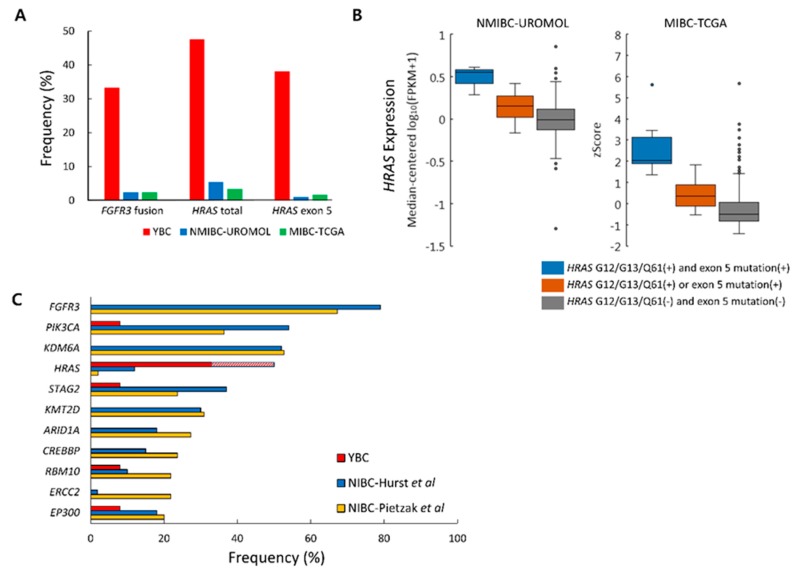
Comparison of young-onset bladder cancer (YBC) with adult bladder cancer (ABC). (**A**) The frequencies of *FGFR3* fusion and *HRAS* mutations in YBC were higher than in adult NMIBC (UROMOL) and muscularis propria-invasive bladder cancer (MIBC) (The Cancer Genome Atlas (TCGA)). (**B**) Synergic effect of *HRAS* G12/G13/Q61 and exon 5 mutations on gene expression observed in adult NMIBC (UROMOL) and MIBC (TCGA). (**C**) We compared the frequencies of somatic point mutations of YBC and two adult genomic studies on NIBC tumors (Pietzak et al. [22] and Hurst et al. [23]), where YBC tumors with paired normal samples (n = 12) and ABC tumors with Ta stage were included (n = 82 in Hurst et al.; n = 55 in Pietzak et al. [22]). Somatic point mutations found in more than 20% of cases in each study are shown. The diagonal pattern of the *HRAS* bar in YBC indicates the exon 5 large deletion.

Although *HRAS* mutation (47.6%) was the most common alteration in YBC, its frequencies were 5.4% and 3.4% in adult NMIBC (UROMOL) and MIBC (TCGA), respectively. *HRAS* exon 5 mutation frequencies were also very low in ABC, at 1.1% (UROMOL) and 1.7% (TCGA) (Tables S2–S4 and Figure 4A) [7,10]. Like YBC, different *HRAS* expression levels according to the *HRAS* mutation pattern were also evident in the ABC data (Figure 4B). The group with concurrent G12/G13/Q61 and exon 5 mutations showed higher *HRAS* expression when compared to groups with only one of those mutations, implying that the two mutations have additive effects on tumorigenesis. A large deletion of *HRAS* exon 5 was not observed in either of the ABC data groups. 

We investigated *HRAS* exon 5 mutations in 9397 primary solid tumors of 24 cancer types from the TCGA. We found that *HRAS* exon 5 mutations were extremely rare, with only two cases present. The two cases were one thymoma and one non-small cell lung cancer, which had 10 and one base deletions within exon 5, respectively (Table S5). Neither splice site mutation nor large deletion of exon 5 was identified. Overall findings suggested that the *HRAS* exon 5 mutation was characteristic of YBC. 

The frequency of the *FGFR3* fusion also differed between YBC and ABC. *FGFR3* fusions have rarely been reported in NMIBC, with one case out of 18 (5.6%) in the Chinese Cancer Genome Consortium (CCGC) study and four cases out of 105 (3.8%) in Pietzak et al. harboring the mutation [22,24]. In the analysis of UROMOL transcriptomic data [7], 11 cases (2%) had the *FGFR3* fusion. 10 cases were *FGFR3-TACC3*, and one was *FGFR3-UBE2K* (Table S6 and Figure 4A). *FGFR3* fusions were also not common in MIBC, being reported in 10 cases out of 408 (2.4%) in TCGA [10], one case out of 24 (4.2%) in CCGC [24], and two cases out of 35 (5.7%) in the study by Ross and colleagues [25].

As for other somatic mutations, frequently mutated genes in adult NIBC cases [22,23] were rarely found in YBC cases (Figure 4C). *FGFR3* was the most commonly mutated gene in adult NIBC, with somatic mutation rates reaching 79% [23]. In contrast, none of the YBC cases showed any *FGFR3* somatic point mutations. The somatic *TP53* mutation, which is the most commonly mutated gene in MIBC, was also not found in YBC. 

### 2.7. No progression to Muscle-Invasive Tumor in ABCs with FGFR3, KRAS, or HRAS Alteration

**Figure 5:**
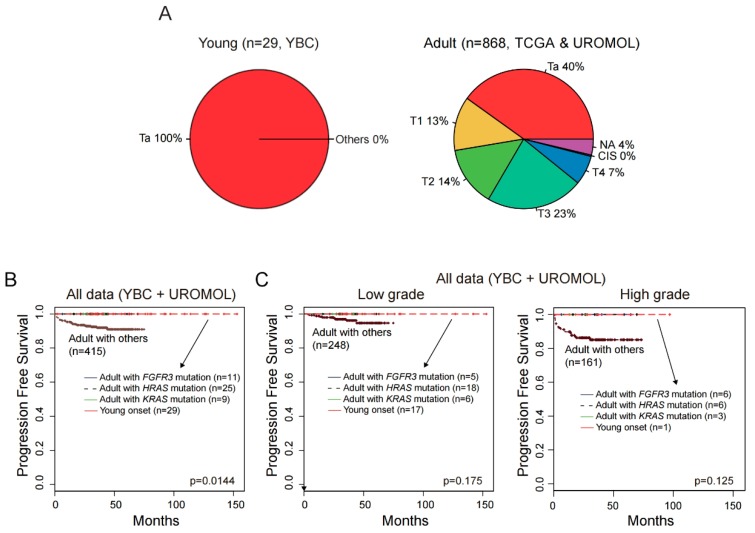
Clinical outcomes of bladder cancer according to genetic alterations. (**A**) All YBCs were non-invasive papillary tumors (Ta stage), but ABC data included various tumor stages. (**B**) All YBCs and ABCs with YBC-like genetic alterations showed no progression to muscle-invasive tumors and better progression-free survival than ABCs with other genetic alterations. (**C**) Progression-free survival based on tumor grade.

Overall, YBC appeared to show better recurrence-free survival than ABC. Nevertheless, it should be noted that all YBC cases were non-invasive papillary tumors (Ta stage), but ABC data included various tumor stages (Figure 5A). When recurrence-free survival was compared after tumor stage and grade matching, no significant difference was found between YBC and ABC (Figure S6). However, no YBC showed progress to a muscle-invasive tumor, while 6.7% (31 out of 460) of ABC (UROMOL data) did. Because the *FGFR3* fusion and *KRAS* and *HRAS* mutations were frequent in YBC, we evaluated whether these genetic alterations affected progression-free survival in ABC. All ABC cases with these genetic alterations showed no progression to muscle-invasive tumors. Moreover, progression-free survivals of YBCs and ABCs with these genetic alterations were significantly better than those of ABCs without the genetic alterations (*p* = 0.0144, Figure 5B). These patterns were also observed in low-grade and high-grade groups and in groups additionally subdivided by stages, albeit without statistical significance (Figure S7 and Figure 5C). These findings indicate that the characteristic genomic features of YBC reflect a good prognosis in bladder cancers.

## 3. Discussion

We report that YBC has low somatic mutation rates (0.3/Mb), which are similar to those of other pediatric tumors (0.37/Mb) [26] in TCGA but much lower than either MIBC or NMIBC in adults. The former is classified as one of the most highly mutated tumors (5.8 mutations/Mb), surpassed only by melanoma (18.4 mutations/Mb) and lung cancer (9.1 mutations/Mb) in TCGA [10].

Genomic alterations in *HRAS* exon 5 have a significant influence on expression and transforming activity. The *HRAS* gene has two stop codons: one in exon 5 and the other in exon 6 (Figure 2). An exon 5 mutation disrupts the stop codon and allows transcription into exon 6, producing *HRAS* proteins with unique C terminal motifs that are essential for the transforming activity [27,28]. According to previous reports, translation into exon 6 results in a marked increase in *HRAS* expression and transforming activity. A subsequent study found concurrent mutations at codon 12 and the exon 5 splicing site in seven out of 67 human bladder cancer cases, showing that the exon 5 splicing site mutation had a greater influence on *HRAS* expression than the codon 12 mutation [29]. Here, we found that the frequency of the exon 5 mutation and the total *HRAS* mutation frequency was greater in YBC than in ABC, which suggests a pivotal role for *HRAS* in YBC oncogenesis.

*FGFR3* is a transmembrane receptor tyrosine kinase that interacts with adaptor proteins containing *GRB2* and activates downstream signaling such as the RAS and MAPK/ERK (Figure S8A) and the PI3K/AKT (Figure S8B) pathways [30]. The latter is also activated by *HRAS*. The transforming potentials of *FGFR3* and *HRAS* are mainly mediated by these pathways. We found that YBC cases with the *HRAS* mutation (Figure S8C) or *FGFR3* fusion (Figure S8D) had a higher upregulation of these pathways than the NMIBC adult cases with such genetic alteration. Therefore, the MAPK/ERK and PI3K/AKT pathways are presumed to play a central role in YBC tumorigenesis.

Importantly, YBC and ABC that harbored YBC-like genetic alterations (*FGFR3* fusions and *KRAS* or *HRAS* mutation) had better progression-free survival than other ABCs. Our results suggest that YBC and ABC with YBC-like putative driver genetic alterations represent indolent bladder tumors that may be managed with less aggressive surveillance strategies. However, this study did not show that the YBC-like mutations were independent prognostic factors. We could not build a multivariate Cox regression model, probably due to the small number of cases and the lack of progression events in cases with those genetic alterations. Therefore, our results should be validated in larger, preferably multinational studies involving a wide range of ethnicity and Ta tumors.

As racial differences may affect cancer-associated genomic bladder mutations, future comparison studies with Asian ABC at Ta stage are necessary. Most of the data used to compare with YBC were generated from Western countries. To the best of our knowledge, only the CCGC study has comprehensive genomic data of Asian ABC [24]. Of the 42 cases with RNA sequencing data in the CCGC study, 18 cases were NMIBC and three of them were Ta stages. This is too small a number for meaningful comparisons with our results, except for one tumor with the *FGFR3* fusion mentioned in the results. 

This study has additional limitations, including its retrospective design and limited analysis of whole exome and RNA. Therefore, the results must be independently validated. 

## 4. Materials and Methods

### 4.1. Patients and Pathologic Diagnosis

Although various age cut-offs like 20, 30, or 40 to 45 years have been used to categorize young patients, a strict age cut-off of 20 years was chosen here [16,31]. As a result, 29 cases of biopsy-proven YBC were collected using a nation-wide search of archived files from the Korean Genitourinary Study Group of the Korean Society of Pathologists. Due to unavailability of tumor tissues (two cases) and poor DNA quality (six cases), molecular analysis was performed in 21 cases. All tumor tissues were transurethral resections of bladder tumor specimens that were formalin-fixed and paraffin-embedded. Among the 21 cases, normal control samples were available in 12 cases. Of those 12 cases, nine had fresh frozen blood and three had normal muscularis propria of the urinary bladder. 

Clinicopathological information, like tumor recurrence and survival, was obtained from patient medical records. All pathologic materials were reviewed by an uropathologust (Y.C.) for diagnostic reassessment and histologic tumor grading according to the 2016 World Health Organization Tumor Classification [1]. Tumor node metastasis (TNM) stage was assigned according to the American Joint Committee on Cancer Staging System, 8th edition [32]. This retrospective study was approved by the institutional review boards of the participating institutions.

### 4.2. Whole Exome Sequencing and Analysis

WES was performed on 21 tumor samples and 12 matched normal samples. Genomic DNA was extracted from tumors and formalin-fixed and paraffin-embedded (FFPE) normal tissue or fresh blood samples using the Maxwell 16 FFPE Plus LEV DNA Purification Kit and the Maxwell 16 LEV Blood DNA Kit (Promega, Mannheim, Germany), respectively. The WES library was constructed using the Agilent SureSelect Human All Exon V5 Kit and sequenced on the Illumina HiSeq2500 to obtain paired-end reads. All WES samples showed uniformly good sequencing quality. The average unique coverage of the samples ranged from 100 to 200× (median: 160×).

Sequencing reads were mapped to the reference human genome (hg19) using the Burrows–Wheeler aligner (v.0.7.12) [33]. PCR duplicate reads were marked and removed using Picard (v1.134). Local realignment around indels and base quality recalibration was carried out using the Genome Analysis Toolkit (GATK v3.8) [34].

Single nucleotide variants (SNVs) were identified using MuTect (v1.1.7) with a Bayesian algorithm, and short indels were identified using the GATK IndelGenotyper (v36.3336) [35,36]. Paired-sample variant calling was applied to 12 tumors cases with matched normal samples to detect somatic variants. For nine cases of tumor-only samples without matched normal samples, we used a reference panel of normal variants that were found at least twice in normal sample calls. Called somatic variants were annotated using ANNOVAR (1Feb2016 release) to obtain information including the population frequency and impact on amino acid changes [37]. For the high-confidence detection of somatic variants, we accepted standard filters of MuTect output and excluded variants that met the following criteria: (1) variant allele count <3 in SNVs or <5 in indels, total allele count <10, variant frequency <0.05 in SNVs or <0.10 in indels; (2) oxoG error in SNVs [38]; (3) located in segmental duplication regions of the human genome [39]; (4) common in the normal population as minor allele frequency >0.01 in the 1000 Genome project [40] and the Exome Aggregation Consortium (ExAC) [41].

The detection of somatic CNA was based on the sequencing coverage ratio of tumor-to-reference samples at the target regions. Data from 12 blood and normal bladder tissue samples were used to compose a normal reference panel. The read count and reads per kilobase per million mapped reads (RPKM) values were calculated for target regions of each sample using CoNIFER (copy number inference from exome reads, v0.2.2) [42]. The median RPKM values of 12 blood and normal bladder samples at the target regions were taken as reference values that were compared to tumor RPKM values. The RPKM values of tumors in the target regions were divided by the corresponding values of the normal reference panel and converted to log2 scale to obtain log2 ratios that were simultaneously evaluated with loss of heterozygosity to detect somatic CNAs. To adjust for the low resolution of WES and detect reliable somatic CNAs, we defined a CNA as a change of log2 (tumor/reference) or a loss of heterozygosity longer than 10 Mb. 

We used DELLY (v0.7.2) to identify structural variations (SVs) including translocations, inversions, and large deletions not detected using an indel caller. These results were annotated using ANNOVAR and filtered using an in-house tool to increase reliability and eliminate artifacts [43]. SV candidates with more than three chimeric reads with a mapping quality of 20 or more and with both breakpoints not located in the intergenic region were considered for further review. Finally, chimeric reads of SV candidates that met the criteria or were located in regions of interest were extracted and manually reviewed using the Integrative Genomics Viewer (v2.3.6) [44].

### 4.3. RNA Sequencing and Analysis

The gene expression altered by genetic alteration was confirmed using RNA sequencing in 11 cases when additional samples were available. RNA was extracted from tumor FFPE tissue using Trizol. Using the Illumina TruSeq RNA Access Library Preparation Kit, an RNA sequencing library was constructed for RNA samples with DV200 values of >30%. The sequencing system was the same as in WES.

STAR (v2.5.3a) and STAR-fusion (v0.8.0) were used to align sequencing reads to human reference (hg19) and detect fusion genes [45]. For the expression analysis, we used RSEM (v1.2.31) to calculate the read counting and fragments per kilobase per million mapped reads (FPKM) values for the Ensemble gene set [46]. The mitogen-activated protein kinase (MAPK)/extracellular-signal-regulated kinase (ERK) and phosphoinositide 3-kinase (PI3K)/AKT scores were defined as the mean of the log10 (median-centered FPKM+1) values of genes belonging to each pathway (Figure S8) [47].

### 4.4. Availability of Data

The expression matrix was deposited in the Gene Expression Omnibus under the accession number GSE133192. All raw data are available for researchers who reasonably request it from the corresponding author with approval of the institutional review boards.

### 4.5. Fusion Breakpoint PCR and Sanger Sequencing 

*HRAS* exon 5 small deletion and structural variations including *HRAS* large deletion and *FGFR3* fusions were confirmed in tumor DNA samples using PCR (Seoul National University) and Sanger sequencing (Macrogen Inc., Seoul, Korea). The PCR primer sequences used are listed in Table S7. 

*HRAS* and *KRAS* single nucleotide variants (SNVs) were confirmed using the same method (Macrogen). All Sanger sequencing results were aligned to the human genome (hg19) using the NCBI Basic Local Alignment Search Tool (https://www.ncbi.nlm.nih.gov/BLAST/) and manually reviewed.

### 4.6. Statistical Analysis

The unequal variance t-test for continuous data and Fisher’s exact test for categorical data were used to compare groups. The threshold for statistical significance was a *p*-value less than 0.05.

### 4.7. Comparative Analysis Between YBC and ABC

For a comparative analysis between YBC and ABC, we retrieved data on NIBC (Hurst et al., Ta, n = 82; Pietzak et al., Ta, n = 55) [22,23], NMIBC (UROMOL study, ≤ T1, n = 460) [7], MIBC (TCGA, Stages II–IV, n = 408) [10], NMIBC and MIBC (Chinese Cancer Genome Consortium (CCGC) study, ≤ T4, n = 42) [24], and metastatic bladder cancer (Ross et al., Stage IV, n = 35) [25] (Table S8). 

WES data from 9397 cases of 24 cancer types from the TCGA data portal were also analyzed. Our own analysis pipeline was used with the UROMOL and TCGA data sets to identify *HRAS* mutations at codons G12/G13/Q61 and exon 5 (splicing site mutation and small/large indels). In addition, we identified the *FGFR3* fusion from the UROMOL data using the same methods applied to the YBC data analysis. 

## 5. Conclusions

YBC had distinct driver genetic alterations such as HRAS mutation and FGFR3 gene fusion and showed good prognosis. YBCs and ABCs with YBC-like mutations showed no progression to muscle-invasive tumors. Therefore, bladder cancer with YBC-like genetic alterations represented an indolent bladder tumor, regardless of age, and may be managed with less aggressive treatment. 

## Supplementary Materials

The following are available online at https://www.mdpi.com/2072-6694/12/2/307/s1. Figure S1: Results of Sanger Sequencing at the *FGFR3* Fusion Breakpoint, Figure S2: Gene Expression Before and After the *FGFR3* Fusion Breakpoint, Figure S3: Detailed Nucleotide and Amino Acid Changes of *HRAS* Deletion, Figure S4: CNAs of Young-Onset Bladder Cancer (YBC), Figure S5: PCA Plots of YBC and the UROMOL Data, Figure S6: Overall Recurrence-Free Survival Rates of YBC and Adult Bladder Cancer (ABC), Figure S7: Progression-Free Survival Rates of YBC and ABC Subdivided by Grade and Stage, Figure S8: MAPK/ERK and PI3K/AKT Pathway Activity in YBC and the UROMOL Data, Table S1: Clinicopathological Characters of the Cases, Table S2: HRAS Mutation Frequencies in YBC, Adult NMIBC (UROMOL), and Adult MIBC (TCGA), Table S3: *HRAS* Mutations in Adult NMIBC (UROMOL), Table S4: *HRAS* Mutations in Adult MIBC (TCGA), Table S5: *HRAS* Exon 5 Mutations in Other TCGA Solid Tumors, Table S6: *FGFR3* Fusions in Adult NMIBC (UROMOL), Table S7: PCR primer Sequences Used, Table S8: Adult Bladder Cancer Studies Used for Comparison with Young-Onset Bladder Cancer (YBC).
