# Supplementary material for: Genomic Landscape of Young-Onset Bladder Cancer and Its Prognostic Implications on Adult Bladder Cancer
Source: Cancers (Basel). 2020 Jan 28;12(2):307. doi: 10.3390/cancers12020307 (PMC7073191; doi:10.3390/cancers12020307)
Supplement: Supplementary file 1 [file cancers-12-00307-s001.zip › cancers-672039-supplement final/Supplementary contents and tables.pdf]

Supplementary Material

Contents:

Supplementary Figure S1. Results of Sanger Sequencing at the *FGFR3* Fusion Breakpoint

Supplementary Figure S2. Gene Expression Before and After the *FGFR3* Fusion Breakpoint

Supplementary Figure S3. Detailed Nucleotide and Amino Acid Changes of *HRAS* Deletion

Supplementary Figure S4. CNAs of Young-Onset Bladder Cancer (YBC)

Supplementary Figure S5. PCA Plots of YBC and the UROMOL Data

Supplementary Figure S6. Overall Recurrence-Free Survival Rates of YBC and Adult Bladder Cancer (ABC)

Supplementary Figure S7. Progression-Free Survival Rates of YBC and ABC Subdivided by Grade and Stage

Supplementary Figure S8. MAPK/ERK and PI3K/AKT Pathway Activity in YBC and the UROMOL Data

Supplementary Table S1. Clinicopathological Characters of the Cases. Supplementary Table S1 is supplied as a separate data sheet.

Supplementary Table S2. *HRAS* Mutation Frequencies in YBC, Adult NMIBC (UROMOL), and Adult MIBC (TCGA)

Supplementary Table S3. *HRAS* Mutations in Adult NMIBC (UROMOL)

Supplementary Table S4. *HRAS* Mutations in Adult MIBC (TCGA)

Supplementary Table S5. *HRAS* Exon 5 Mutations in Other TCGA Solid Tumors

Supplementary Table S6. *FGFR3* Fusions in Adult NMIBC (UROMOL)

Supplementary Table S7. PCR primer Sequences Used

Supplementary Table S8. Adult Bladder Cancer Studies Used for Comparison with Young-Onset Bladder Cancer (YBC)

Supplementary Table S2. *HRAS* Mutation Frequencies in YBC, Adult NMIBC (UROMOL), and Adult MIBC (TCGA).

	**YBC (n = 21)**		**NMIBC (n = 460)**		**MIBC (n = 408)**	
	n	%	n	%	n	%
G12/G13/Q61 mutation	6	28.6	23	4.8	13	3.2
Exon 5 small indel	4 (4) *	19.0	5 (3)*	1.1	4 (3) *	1
Exon 5 splice site mutation	0	0	N/A	N/A	3 (3) *	0.7
Exon 5 large deletion	4	19.0	0	0	0	0
Total	10	47.6	25	5.4	14	3.4

* The number of samples with concurrent G12/G13/Q61 and exon 5 mutations.

Supplementary Table S3. *HRAS* Mutations in Adult NMIBC (UROMOL).

ID	stage	chromosome	position	REF	ALT	*HRAS* mutation
U0046	T1	11	534286	C	G	G13R
U0060	Ta	11	533874	T	C	Q61R
U0094	Ta	11	534288	C	A	G12V
U0149	Ta	11	533301	TGGGTCCCGGG	T	exon 5 indels
U0204	Ta	11	533874	T	C	Q61R
U0214	T1	11	534286	C	G	G13R
U0219	Ta	11	533874	T	C	Q61R
U0334	T1	11	534288	C	T	G12D
U0334	T1	11	534285	C	A	G13V
U0350	Ta	11	533874	T	C	Q61R
U0461	Ta	11	534285	C	A	G13V
U0496	Ta	11	533301	TGGGTCCCGGG	T	exon 5 indels
U0496	Ta	11	533874	T	C	Q61R
U0573	T1	11	533311	GGGGTCCCAGA	G	exon 5 indels
U0573	T1	11	533874	T	A	Q61L
U0590	Ta	11	533875	G	T	Q61K
U0642	Ta	11	534286	C	G	G13R
U0670	T1	11	533874	T	C	Q61R
U0847	Ta	11	533874	T	C	Q61R
U0915	Ta	11	534286	C	G	G13R
U0973	Ta	11	533301	TGGGTCCCGGG	T	exon 5 indels
U1031	Ta	11	533874	T	C	Q61R
U1050	Ta	11	533874	T	C	Q61R
U1150	Ta	11	533357	C	CGC	exon 5 indels
U1150	Ta	11	533874	T	A	Q61L
U1233	T1	11	534286	C	G	G13R
U1270	Ta	11	533874	T	C	Q61R
U1374	T1	11	533874	T	A	Q61L
U1413	Ta	11	533874	T	C	Q61R

Supplementary Table S4. *HRAS* Mutations in Adult MIBC (TCGA).

**chromosome**	**position**	**REF**	**ALT**	***HRAS* mutation**
11	534285	C	T	G13D
11	533344	GCCAGAGCGGCTGCCCTGTGTCAAGGGAGAGGGTCAGTGAGT	G	exon 5 indels
11	534288	C	T	G12D
11	533276	C	G	exon 5 splice site mutation
11	533874	T	A	Q61L
11	533308	CG	C	exon 5 indels
11	534285	C	A	G13V
11	533276	C	A	exon 5 splice site mutation
11	534289	C	T	G12S
11	533305	TCCCGGGGGGTCCCAGAGGGTCCCGGAGCTGGAGCTAGAGCCAGA	TG	exon 5 indels
11	534286	C	G	G13R
11	533875	G	T	Q61K
11	533276	C	T	exon 5 splice site mutation
11	533311	GGGGTCCCAGA	G	exon 5 indels
11	533874	T	A	Q61L
11	534288	C	T	G12D
11	534286	C	G	G13R
11	533874	T	A	Q61L
11	533875	G	T	Q61K
11	533875	G	T	Q61K

Supplementary Table S5. *HRAS* Exon 5 Mutations in Other TCGA Solid Tumors.

	Number (n) of tested samples	*HRAS* exon 5 mutation (n)	Chromosom e	Position	REF	ALT	*HRAS* mutation
Adrenocortical carcinoma	92	0					
Breast cancer	1044	0					
Cervical cancer	305	0					
Colorectal cancer	590	0					
Endometrial cancer	599	0					
Esophagogastric cancer	625	0					
Germ cell tumor	150	0					
Glioma	906	0					
Head and neck cancer	510	0					
Hepatobiliary cancer	471	0					
Lung cancer	989	1	11	533315	TC	T	exon 5 indels
Melanoma	183	0					
Mesothelioma	23	0					
Miscellaneous neuroepithelial tumor	33	0					
Nerve sheath tumor	9	0					
Non-Hodgkin lymphoma	43	0					
Ovarian cancer	441	0					
Pancreatic cancer	183	0					
Papillary thyroid cancer	496	0					
Pheochromocytoma	147	0					
Prostate cancer	498	0					
Renal cell carcinoma	693	0					
Soft tissue sarcoma	246	0					
Thymic tumor	121	1	11	533301	TGGGTCCCGGG	T	exon 5 indels

Supplementary Table S6. *FGFR3* Fusions in Adult NMIBC (UROMOL).

ID	stage	Fusion
U0090	T1	*FGFR3--TACC3*
U0401	T1	*FGFR3--TACC3*
U0012	Ta	*FGFR3--TACC3*
U1276	Ta	*FGFR3--TACC3*
U1277	Ta	*FGFR3--TACC3*
U0430	Ta	*FGFR3--TACC3*
U0450	Ta	*FGFR3--TACC3*
U0777	T1	*FGFR3--TACC3*
U0130	Ta	*FGFR3--UBE2K*
U1002	Ta	*FGFR3--TACC3*
U0326	Ta	*FGFR3--TACC3*

Supplementary Table S7. PCR Primer Sequences Used.

ID	mutation type	primer1	primer2
T1	*FGFR3* fusion	GCAGGAGTACCTGGACCTGT	ATTGGGTTACTCACACGGGC
T2	*FGFR3* fusion	ACTGGTCCCCAACAATGTGA	CCATGACCGCCTTAGAATGT
T3	*FGFR3* fusion	GACCGTGTCCTTACCGTGAC	TTCCCTCAGACCACACACAA
T4	*FGFR3* fusion	CAGCAGGAGTACCTGGACCT	GCAGAAACCAGCAAATCCAT
T5	*FGFR3* fusion	CAGCAGGAGTACCTGGACCT	CCACAACAATCATACAGGGAGA
T6	*FGFR3* fusion	AGCAGGAGTACCTGGACCTG	GGGGTCTCTGAAGCTCTCCT
T8	*HRAS* large del	GTGGGGAACAAGTGTGACCT	ATCTCACGCACCAACGTGTA
T9	*HRAS* large del	GCTACGGCATCCCCTACAT	GACACTCTGGGGACAAGAGG
T10	*HRAS* large del	GCTACGGCATCCCCTACAT	GACACTCTGGGGACAAGAGG
T12	*HRAS* small del	CTCACTGACCCTCTCCCTTG	AGCTCTCCCCAAGGACCTC
T14	*HRAS* small del	CTCACTGACCCTCTCCCTTG	AGCTCTCCCCAAGGACCTC
T15	*HRAS* small del	CTCACTGACCCTCTCCCTTG	AGCTCTCCCCAAGGACCTC

Supplementary Table S8. Adult Bladder Cancer Studies Used for Comparison with Young-Onset Bladder Cancer (YBC).

Study	Tumor stage	n	Data type
UROMOL study	NMIBC (≤ T1)	460	RNA sequencing
Hurst et al	NIBC (Ta)	82	WES (N = 24) + Target sequencing (N = 58)
TCGA	MIBC (≥ Stage II)	408	WES + RNA sequencing
Pietzak et al	NIBC (Ta)	55	Target sequencing
CCGC study	NMIBC (n = 18) + MIBC (n = 24)	42	RNA sequencing
Ross et al	Metastatic bladder cancer (Stage IV)	35	Target sequencing

WES, whole exome sequencing.
