# Supplementary material for: Genomic Landscape of Young-Onset Bladder Cancer and Its Prognostic Implications on Adult Bladder Cancer
Source: Cancers (Basel). 2020 Jan 28;12(2):307. doi: 10.3390/cancers12020307 (PMC7073191; doi:10.3390/cancers12020307)
Supplement: Supplementary file 1 [file cancers-12-00307-s001.zip › cancers-672039-supplement final/Supplementary_figures.pdf]

Supplementary Figure S1. Results of Sanger Sequencing at the *FGFR3* Fusion Breakpoint. The fusion breakpoint found in NGS was confirmed by bidirectional PCR and Sanger sequencing using tumor DNA.

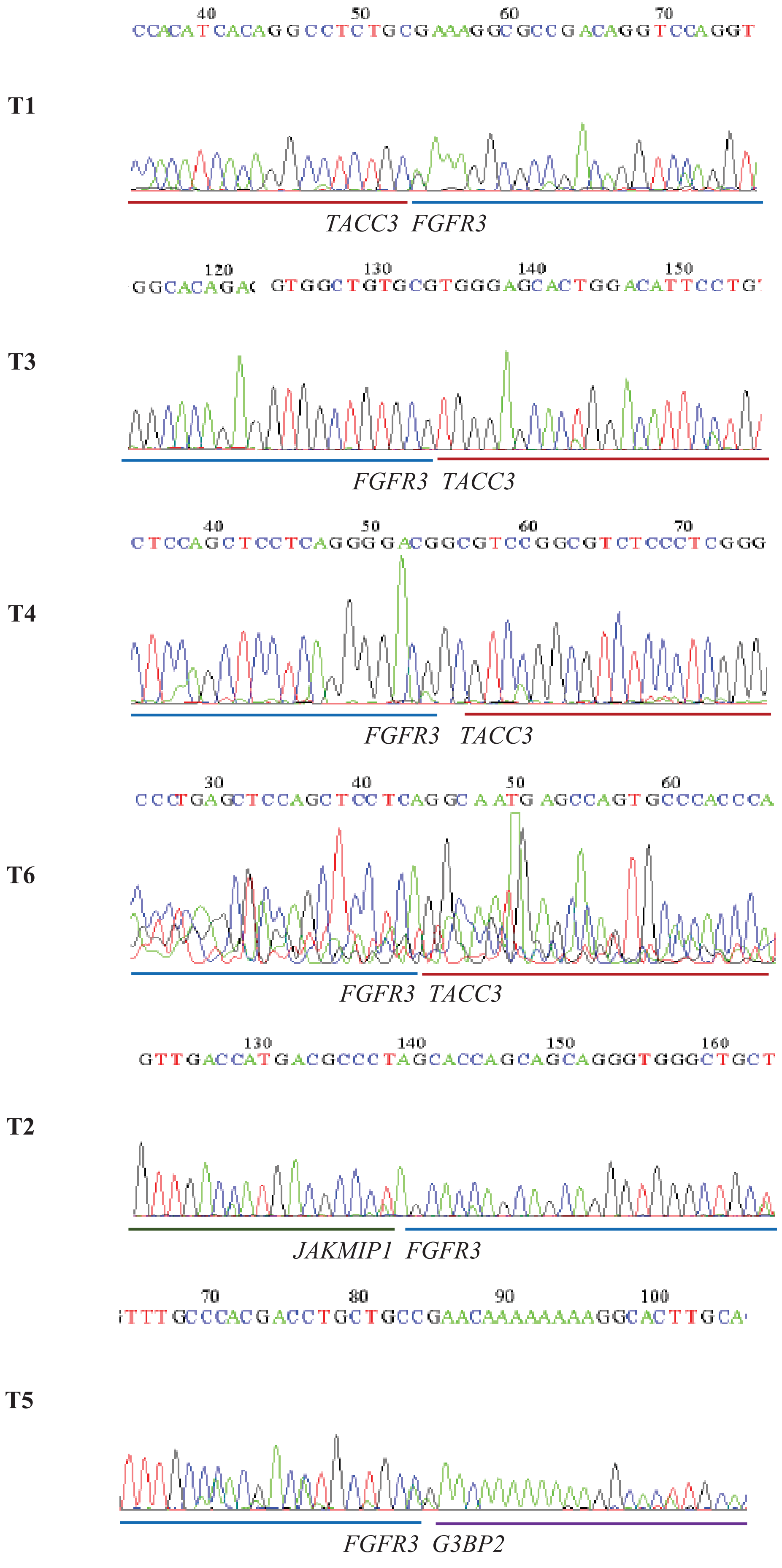

Supplementary Figure S2. Gene Expression Before and After the *FGFR3* Fusion Breakpoint. Y-axis is read count per base pair.

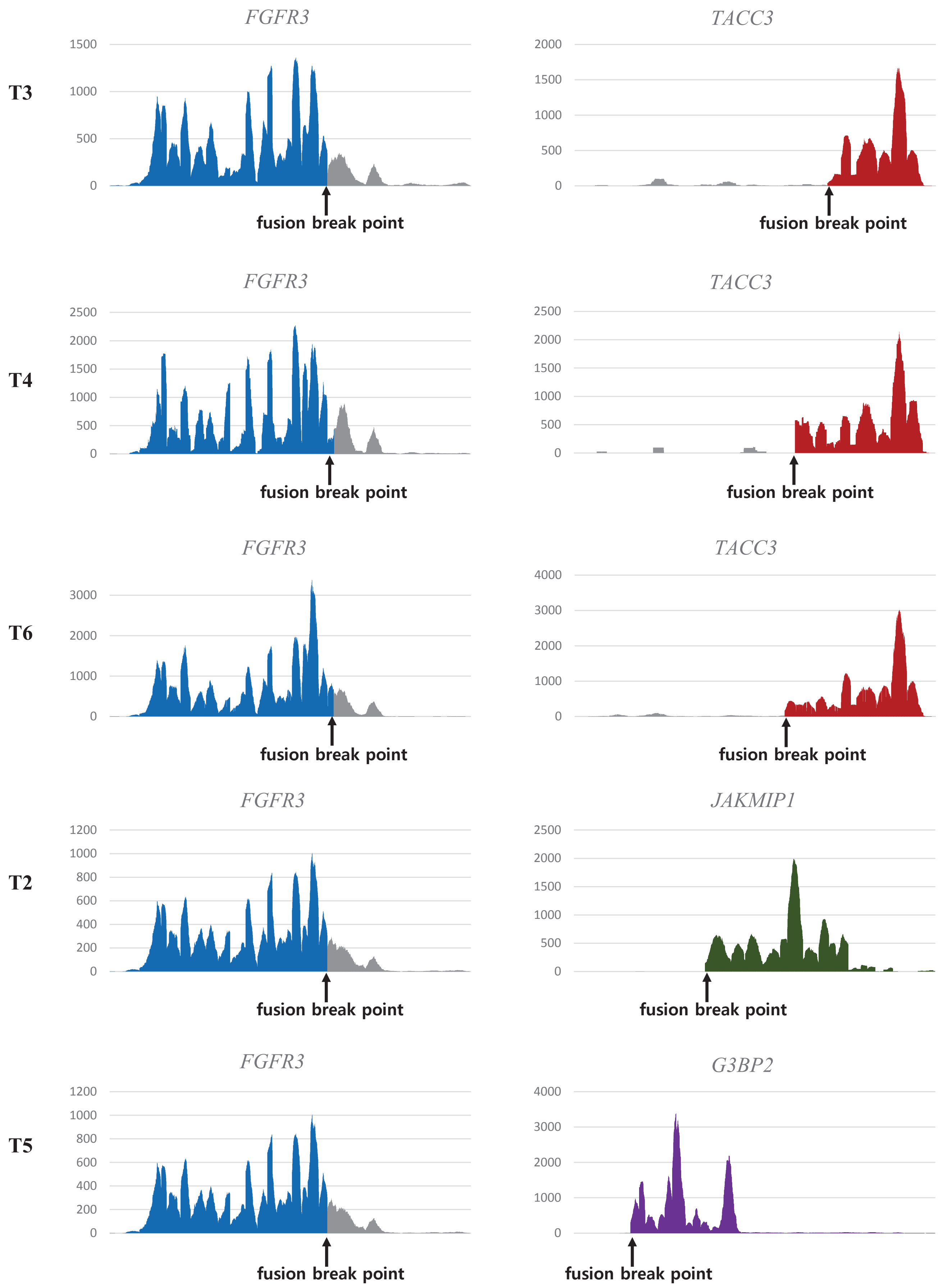

Supplementary Figure S3. Detailed Nucleotide and Amino Acid Changes in *HRAS* Deletion.

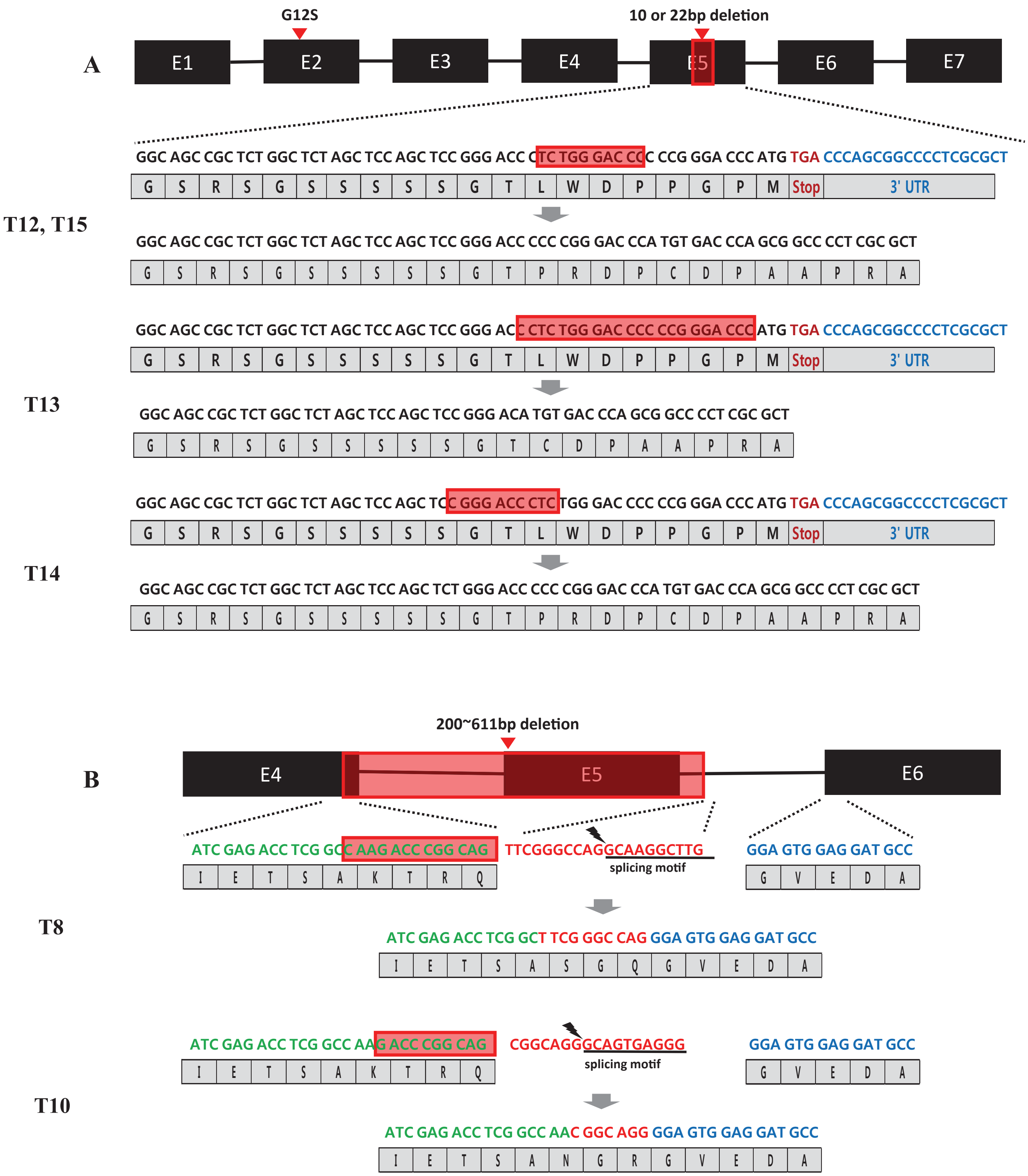

Supplementary Figure S4. CNAs of Young-Onset Bladder Cancer (YBC). Upper and lower panel represent log2 (tumor/reference) ratio and B allele frequency of each tumor.

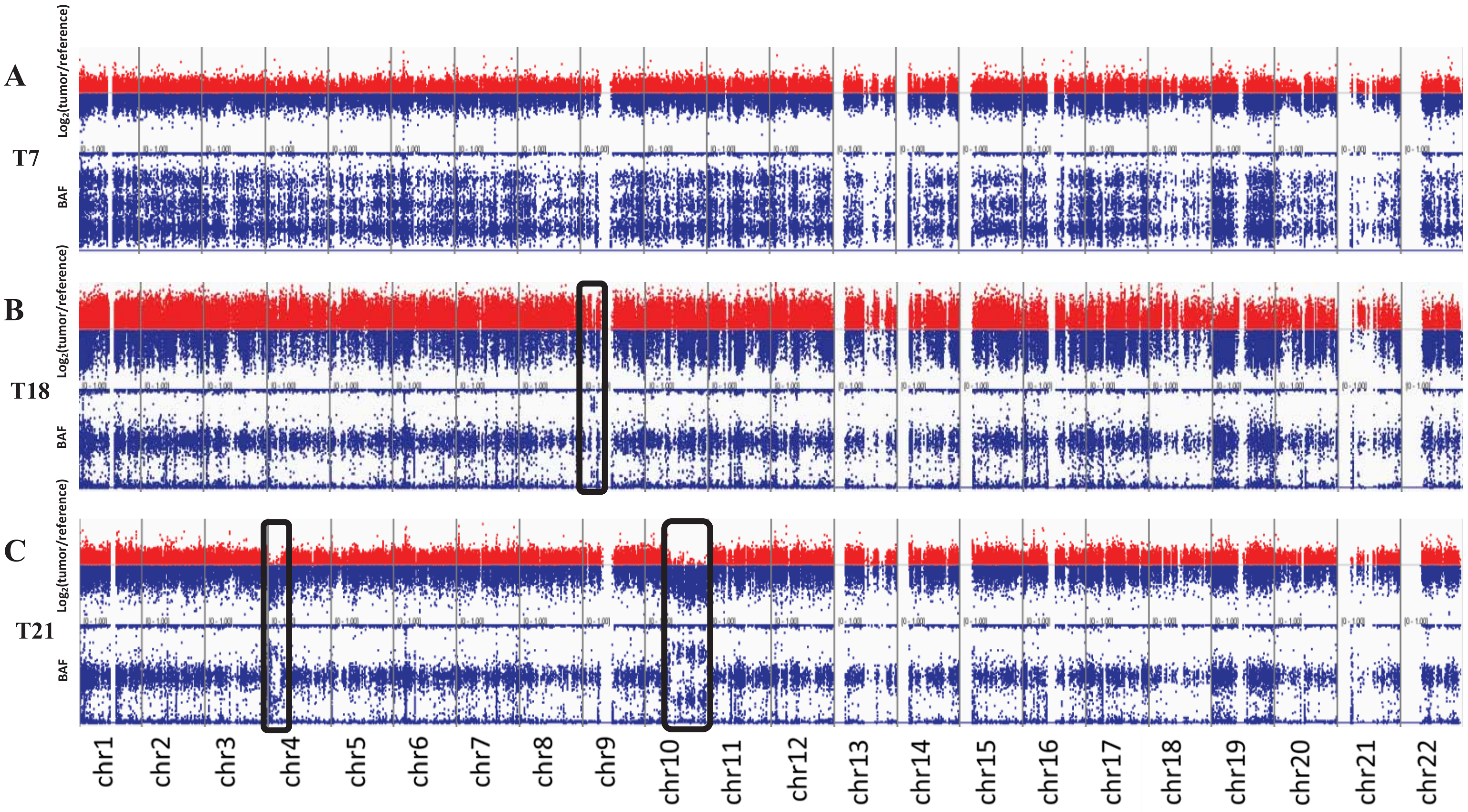

Supplementary Figure S5. PCA Plots of YBC and the UROMOL Data. PCA was calculated using the 117 genes from Fig. 3A.

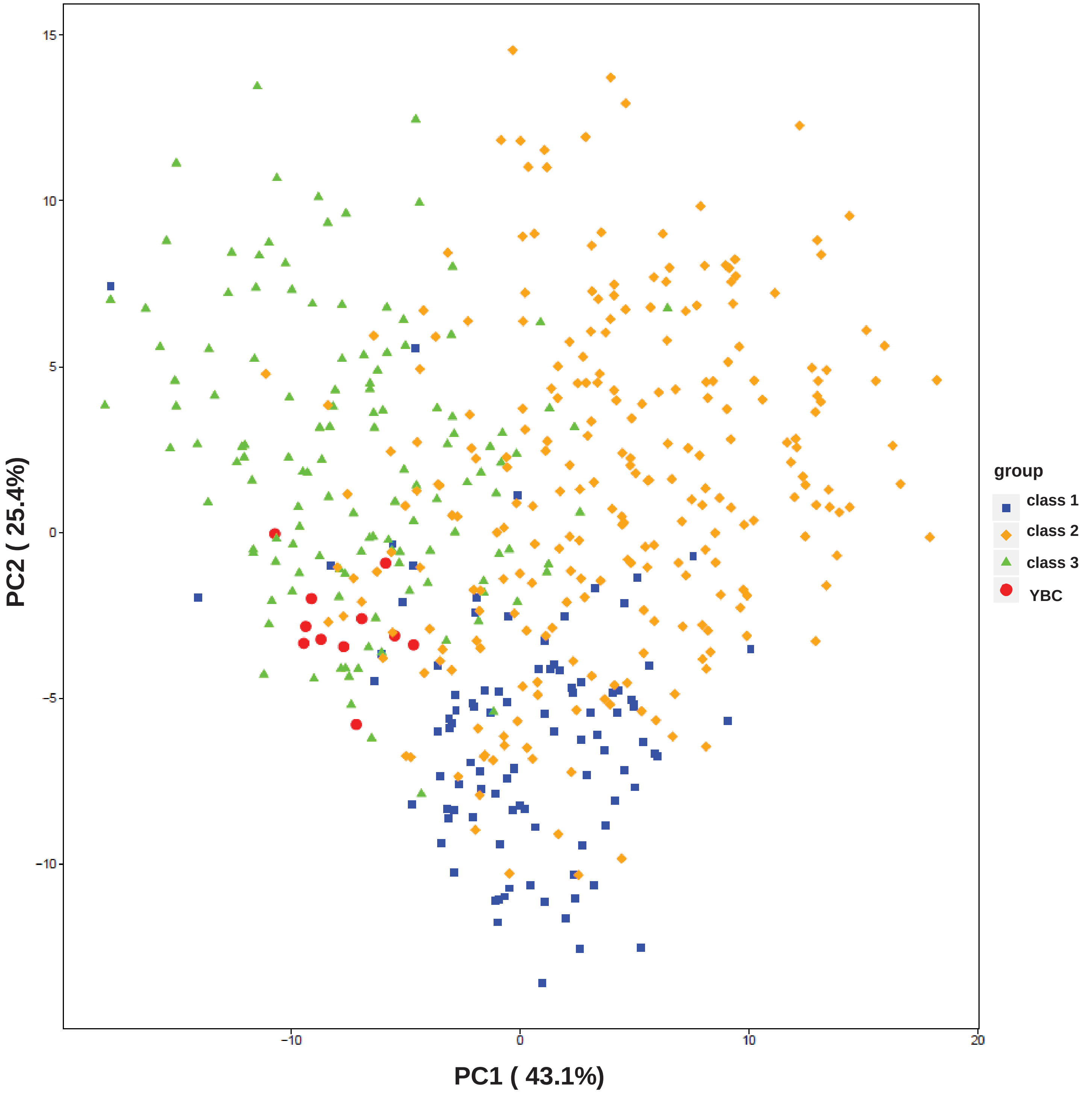

Supplementary Figure S6. Overall Recurrence-Free Survival Rates of YBC and Adult Bladder Cancer (ABC).

YBC showed better recurrence-free survival rates than ABC. When the survivals were compared after tumor stage and grade matching, there was no significant difference between YBC and ABC.

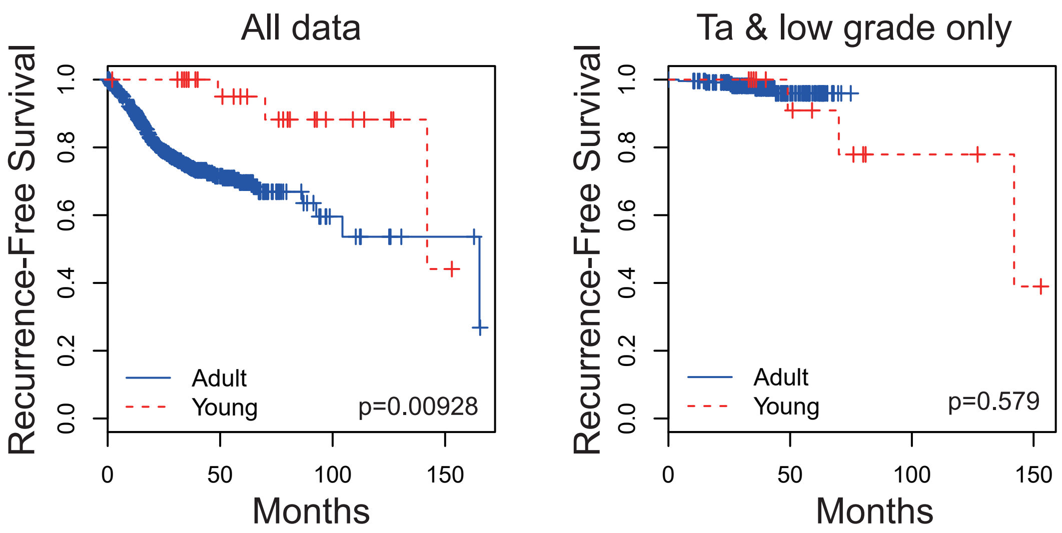

Supplementary Figure S7. Progression-Free Survival Rates of YBC and ABC Subdivided by Grades and Stages.

YBC and ABC with *FGFR3* fusion and *KRAS/HRAS* mutation showed better progression-free survival rates than ABC without these genetic alterations.

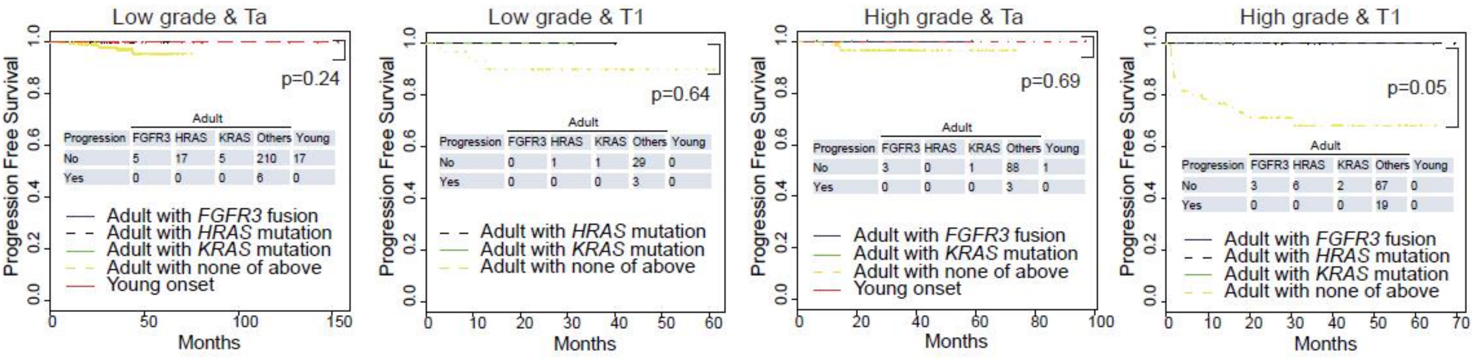

Supplementary Figure S8. MAPK/ERK and PI3K/AKT Pathway Activity in YBC and the UROMOL data.

Expression profiles of 52 genes in the MAPK/ERK pathway (A) and 9 genes in the PI3K/AKT pathway (B) in the whole YBC and UROMOL samples are depicted as a heatmap. FGFR3 and HRAS gene expression level and MAPK/ERK and PI3K/AKT scores in samples with HRAS mutation (C) and FGFR3 fusion (D) are shown as box plots.

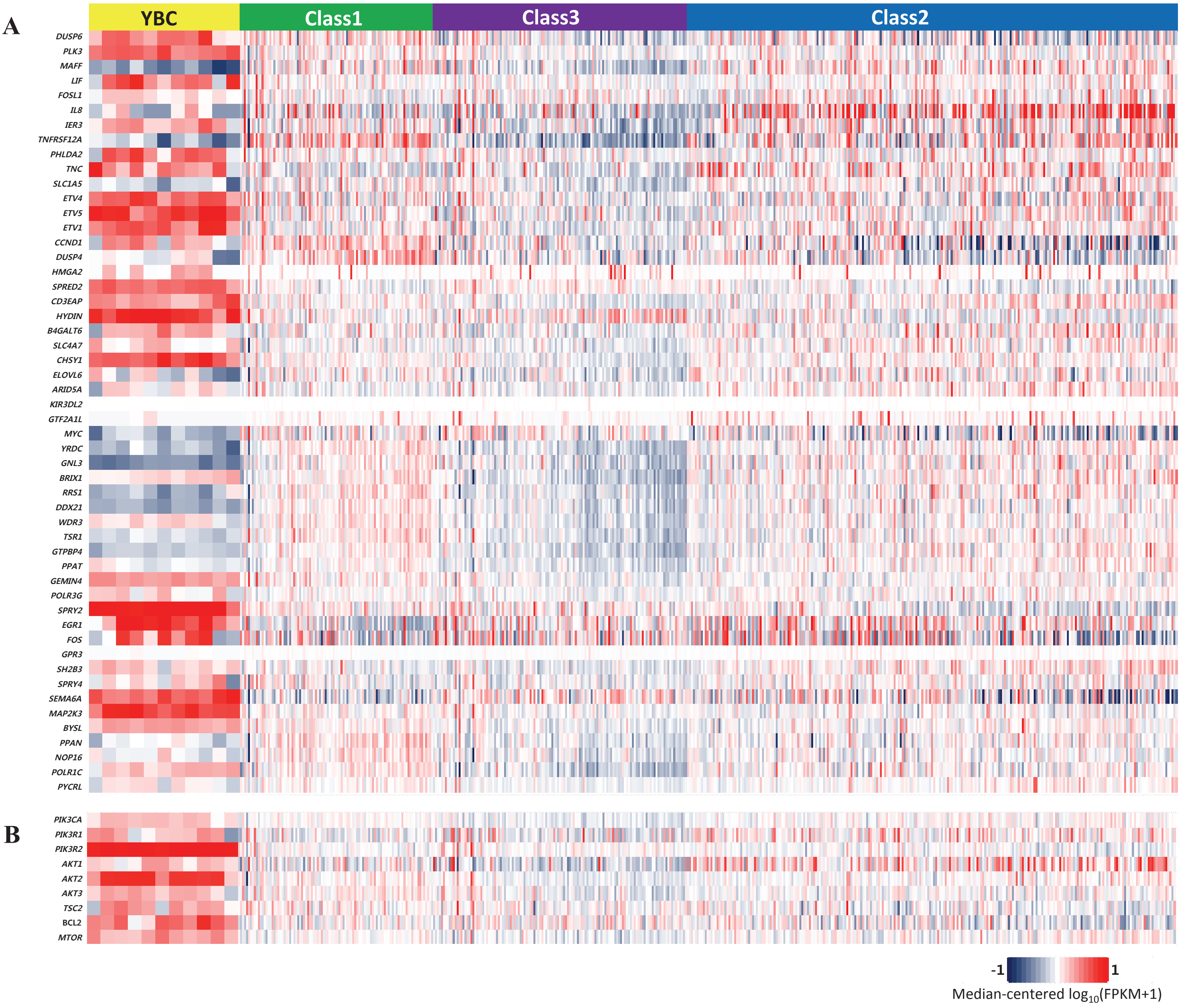

C

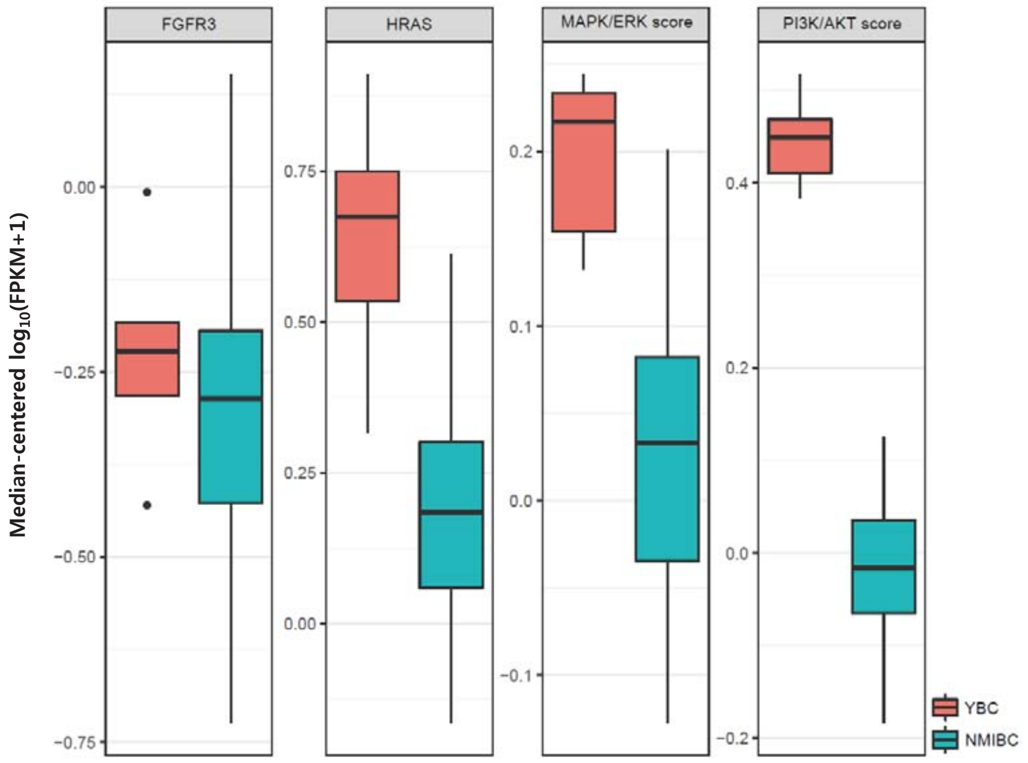
FGFR3
HRAS
MAPK/ERK score
PI3K/AKT score
Median-centered log10(FPKM+1)
0.00
−0.25
−0.50
−0.75
0.75
0.50
0.25
0.00
0.2
0.1
0.0
−0.1
0.4
0.2
0.0
−0.2
YBC
NMIBC

D

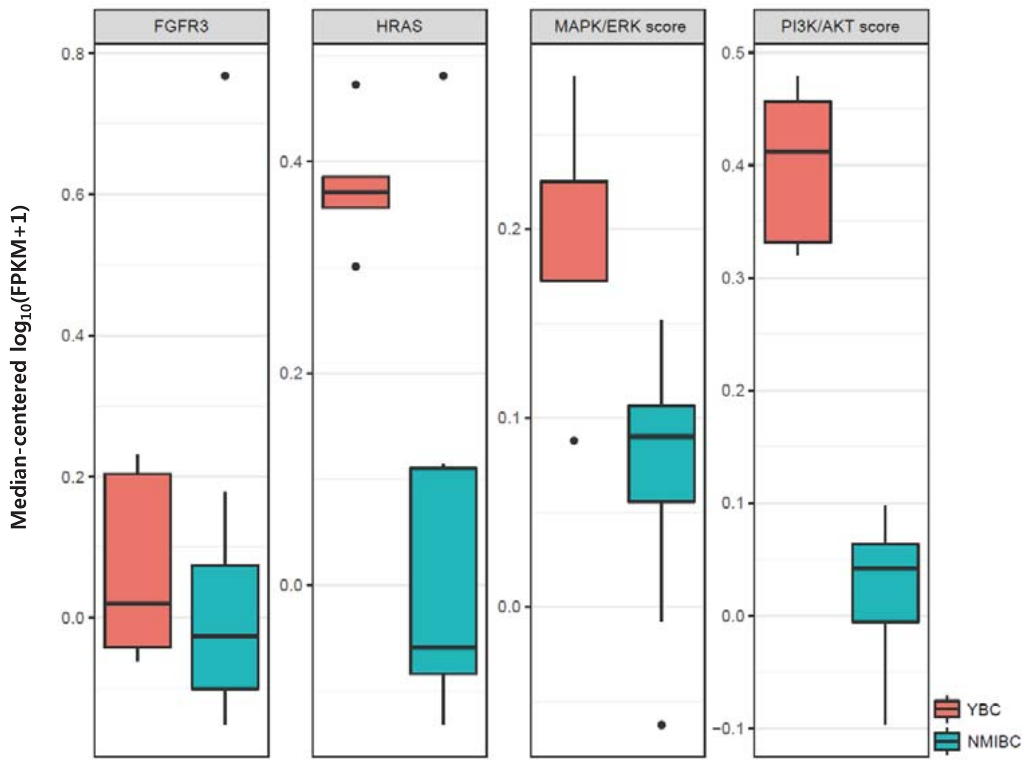
FGFR3
HRAS
MAPK/ERK score
PI3K/AKT score
Median-centered log10(FPKM+1)
0.8
0.6
0.4
0.2
0.0
0.4
0.2
0.0
0.2
0.1
0.0
0.5
0.4
0.3
0.2
0.1
0.0
−0.1
YBC
NMIBC
